# Orientation of Temporal Interference for Non-invasive Deep Brain Stimulation in Epilepsy

**DOI:** 10.3389/fnins.2021.633988

**Published:** 2021-06-07

**Authors:** Florian Missey, Evgeniia Rusina, Emma Acerbo, Boris Botzanowski, Agnès Trébuchon, Fabrice Bartolomei, Viktor Jirsa, Romain Carron, Adam Williamson

**Affiliations:** ^1^Aix-Marseille Université, Inserm, Institut de Neurosciences des Systèmes (INS) UMR_S 1106, Marseille, France; ^2^Department of Functional and Stereotactic Neurosurgery, Timone University Hospital, Marseille, France; ^3^Laboratory of Organic Electronics, Linköping University, Norrköping, Sweden

**Keywords:** electrical stimulation, cortex, hippocampus, seizures, epilepsy, temporal interference

## Abstract

In patients with focal drug-resistant epilepsy, electrical stimulation from intracranial electrodes is frequently used for the localization of seizure onset zones and related pathological networks. The ability of electrically stimulated tissue to generate beta and gamma range oscillations, called rapid-discharges, is a frequent indication of an epileptogenic zone. However, a limit of intracranial stimulation is the fixed physical location and number of implanted electrodes, leaving numerous clinically and functionally relevant brain regions unexplored. Here, we demonstrate an alternative technique relying exclusively on non-penetrating surface electrodes, namely an orientation-tunable form of temporally interfering (TI) electric fields to target the CA3 of the mouse hippocampus which focally evokes seizure-like events (SLEs) having the characteristic frequencies of rapid-discharges, but without the necessity of the implanted electrodes. The orientation of the topical electrodes with respect to the orientation of the hippocampus is demonstrated to strongly control the threshold for evoking SLEs. Additionally, we demonstrate the use of Pulse-width-modulation of square waves as an alternative to sine waves for TI stimulation. An orientation-dependent analysis of classic implanted electrodes to evoke SLEs in the hippocampus is subsequently utilized to support the results of the minimally invasive temporally interfering fields. The principles of orientation-tunable TI stimulation seen here can be generally applicable in a wide range of other excitable tissues and brain regions, overcoming several limitations of fixed electrodes which penetrate tissue and overcoming several limitations of other non-invasive stimulation methods in epilepsy, such as transcranial magnetic stimulation (TMS).

## Introduction

During presurgical evaluation, patients suffering from focal drug-resistant epilepsy often require invasive recordings using stereo-electroencephalography (SEEG), involving the implantation of numerous electrodes in different brain regions for the electrophysiological monitoring of seizure onset and the subsequent localization of an epileptogenic zone (EZ) (Bartolomei et al., 2008; Koessler et al., 2010). Electrical stimulation from the intracranial electrodes is often necessary to help define an EZ, with electrophysiological discharges and seizures triggered by different frequencies of stimulation (Munari et al., 1993; Guye et al., 2006). In general, pathological discharges from the EZ are characterized by several biomarkers, primarily the generation of high-frequency oscillations in the beta/gamma range, classically referred to as rapid-discharges (Allen et al., 1992; Bartolomei et al., 2001; Wendling et al., 2003). Although currently performed with invasive SEEG, less-invasive methods capable of evoking such discharges and seizures during the localization of EZ tissue would be highly interesting as positive surgical outcomes are well-correlated with the removal of tissue regions which generate such high-frequency oscillations (Alarcon et al., 1995; Lagarde et al., 2016; Frauscher et al., 2017; Oderiz et al., 2019).

Interestingly, temporal interference stimulation was only recently introduced, in Grossman et al. (2017). The concept of TI stimulation exploits physiological properties of neurons, namely that the neuronal membranes filter electrical signals of frequencies more than 1 kHz, limiting depolarization and stimulation properties above these frequencies (Hutcheon and Yarom, 2000; Mirzakhalili et al., 2020). In our work presented here, we show that a crucial part of TI stimulation is the electrode orientation – not simply to move the spot of focal stimulation – but more importantly to move the orientation of electric field of the TI with respect to the orientation of the underlying structure to be stimulated. For classic TI, two electric fields are used with each field having a slightly different frequency, *f1* and *f2* = *f1* + Δ*f*, where f1 is selected above the threshold to evoke electrophysiological activity, for example 1200 Hz, and Δ*f* is selected at a frequency typically used to evoke activity, for example 50 Hz (Musto et al., 2009; Cela et al., 2019). The two electric fields of frequency 1200 and 1250 Hz create a low-frequency envelope of 50 Hz. Classically, electrode references have been placed in the chest of animals. However, this allows field lines to pass relatively arbitrarily through subcortical structures such as the hippocampus.

**FIGURE 1:**
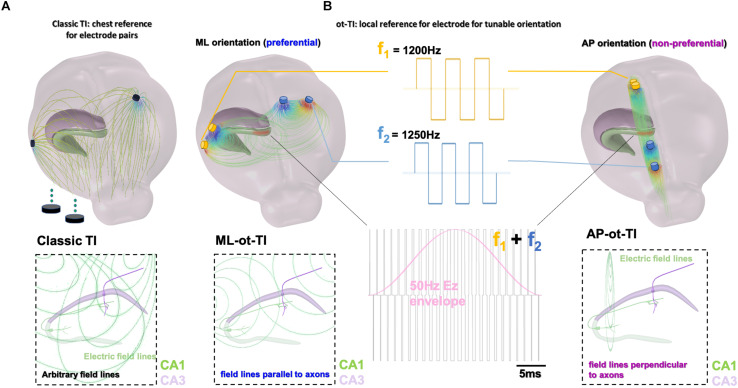
Orientation-tunable Temporally Interfering Electric Fields (ot-TI). **(A)** Classic TI, using electrode pairs with references in the chest, a reliable method to non-invasively stimulate. However, field lines are arbitrary with respect to sub-cortical structures. **(B)** ot-TI, using electrode pairs with local references for controlled-orientation of electric field lines. Here, two perpendicular arrangements of ot-TI are shown, ML with field lines parallel to the alignment of axons in the hippocampus from CA3 to CA1, and AP with field lines perpendicular to the same axons. Classic TI stimulation is performed with sine waves, however, envelopes can also be created with other wave forms, for example square waves as seen here. These two configurations are compared experimentally to determine the minimally invasive efficacy of ot-TI to generate beta/gamma range oscillations and SLEs in the mouse hippocampus.

In the work presented here, we show that it is possible to preferentially evoke activity by exploiting the orientation of the field lines with respect to the subcortical anatomy based on the configuration of the TI electrode pairs. In Figure 1, we utilize local references and orient electrode pairs with respect to axon tracts, the Schafer collaterals, of the CA field. Using the method of orientation-tunable Temporal Interference (ot-TI), we then evoke seizure-like events (SLEs) having the characteristic frequencies of rapid-discharges. In the configuration shown here, we create electric fields parallel (medio-lateral, ML) and perpendicular (anterior-posterior, AP) to the axons in the mouse hippocampus. Furthermore, as shown in Figure 1B and Supplementary Figure 1, it is not necessary to exclusively utilize sine waves for stimulation, a critical detail as clinical stimulation equipment most often uses square waves.

The method presents several clear advantages over classical brain stimulation and potential future applications in presurgical evaluation and treatment of human epilepsy. Our method of ot-TI can explore brain tissue, including sensitive regions previously unavailable for direct implantation, in a minimally invasive way using electrodes on the cortex surface. Points of focal stimulation at significant distances below the cortex surface are created by envelopes of interacting electric fields applied by the surface electrodes to evoke SLEs in the hippocampus. The orientation of the electric field, as defined by the orientation of the surface electrodes, determines the effectiveness and the threshold of stimulation necessary to evoke activity (Lian et al., 2003; Kabakov et al., 2012)200. Our method of ot-TI provides a distinct advantage over transcranial magnetic stimulation (TMS). TMS is the other key non-invasive method for the localization of functional brain areas before epilepsy surgery (Säisänen et al., 2010) and for the localization of the EZ (Vitikainen et al., 2009) but stimulation deep below the cortex is not available with TMS. Additionally, it has been difficult to enhance and evoke seizures with TMS where success has been in no more than 2.8% of cases (Schrader et al., 2004). Indeed, Temporal interference stimulation could provide several key advantages in epilepsy, for foci localization, evoking seizures, and deeper functional brain mapping (Deng et al., 2013).

## Results

### Creating SLEs Using TI in the Mouse Hippocampus

**FIGURE 2:**
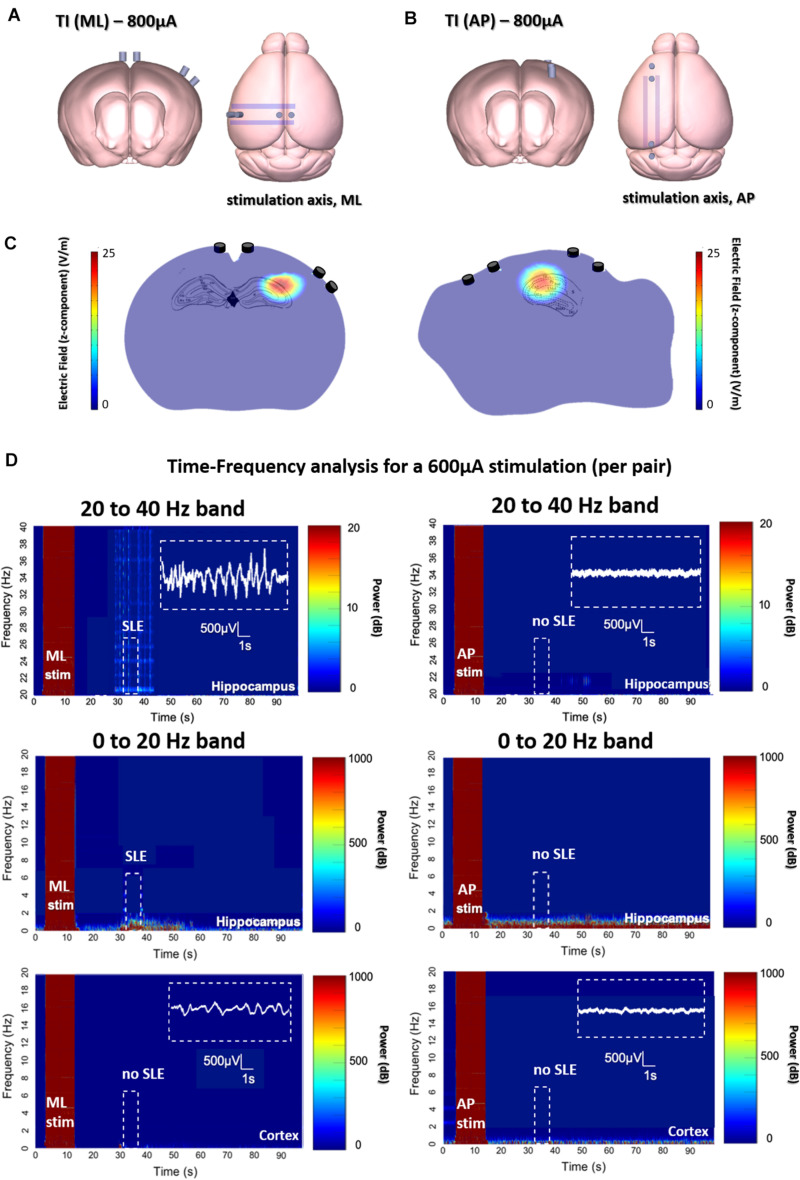
Evoked SLEs in the hippocampus of freely moving mice. **(A,B)** Depict the two ot-TI stimulation axes with electrodes to-scale. **(C)** Using Finite-element method simulation, the associated hotspots (maximum envelopes of electric field) are seen in the hippocampus, with the maximum amplitude placed at the CA3-CA1 junction as planned in the protocol. For ML/AP orientations, the electric field lines are parallel/perpendicular to the Schaffer collaterals. **(D)** Real biologically evoked SLEs in mice using ot-TI. For the ML orientation (left panels), parallel to the axons of the hippocampus, the shape of the evoked activity, the associated increase in frequencies of the beta/gamma range (20–40 Hz), and observed behavioral seizures demonstrate a classic SLE, however, it is the first minimally invasive evoked SLE by using TI. The AP orientation (right panels), perpendicular to the axons of the hippocampus, showed no evoked SLEs, and for 8 mice only one showed a stage 4 behavioral seizure on the Racine scale with a threshold above 900 μA. Placement of recording electrodes in Supplementary Figure 6. Time-Frequency plots and evoked SLEs are shown for 1 representative ML mouse and 1 representative AP mouse from *n* = 8 groups. Group statistics regarding events are shown in subsequent figures.

We created SLEs in the mouse hippocampus using both TI and implantable electrode protocols. The target of stimulation was the border of CA3 and CA1 (detailed coordinates in the section “Material Methods”), well-below the cortex surface. As seen in Figures 2A–C for the two orientations of ot-TI, the maximum envelope is placed using Finite-element method simulations at the same location in the hippocampus, namely the border of the CA3 to CA1. Although the maximum is at the same point, the electric field lines are parallel (for ML) or perpendicular (for AP) to the axons of the hippocampus, the Schaffer Collaterals (SCs). In Figure 2D, for all ML mice, the shape of the evoked activity, the increase in the frequency range of beta/gamma (20–40 Hz), and the behaviorally observed seizures were all consistent with a classically described stimulation-induced focal seizure of the hippocampus, however, this is the first minimally invasive evoked SLE using the method of TI. All of the 8 ML mice exhibited a behavioral seizure 4 on Racine scale (overall threshold of 700 μA per electrode pair) correlated with an electrophysiological SLE.

In Figure 2D for AP mice, interesting things happen when the point of maximum envelope remains at the same coordinates, but the electric field is turned perpendicular to the axons of the hippocampus in a second group of mice. Clearly, no SLE activity is evoked with the AP orientation, although the amplitude of the envelope and its location are the same for both ML and AP. It is therefore necessary to increase the intensity of stimulation.

### Implication of Field Lines Orientation in Seizures Induction

**FIGURE 3:**
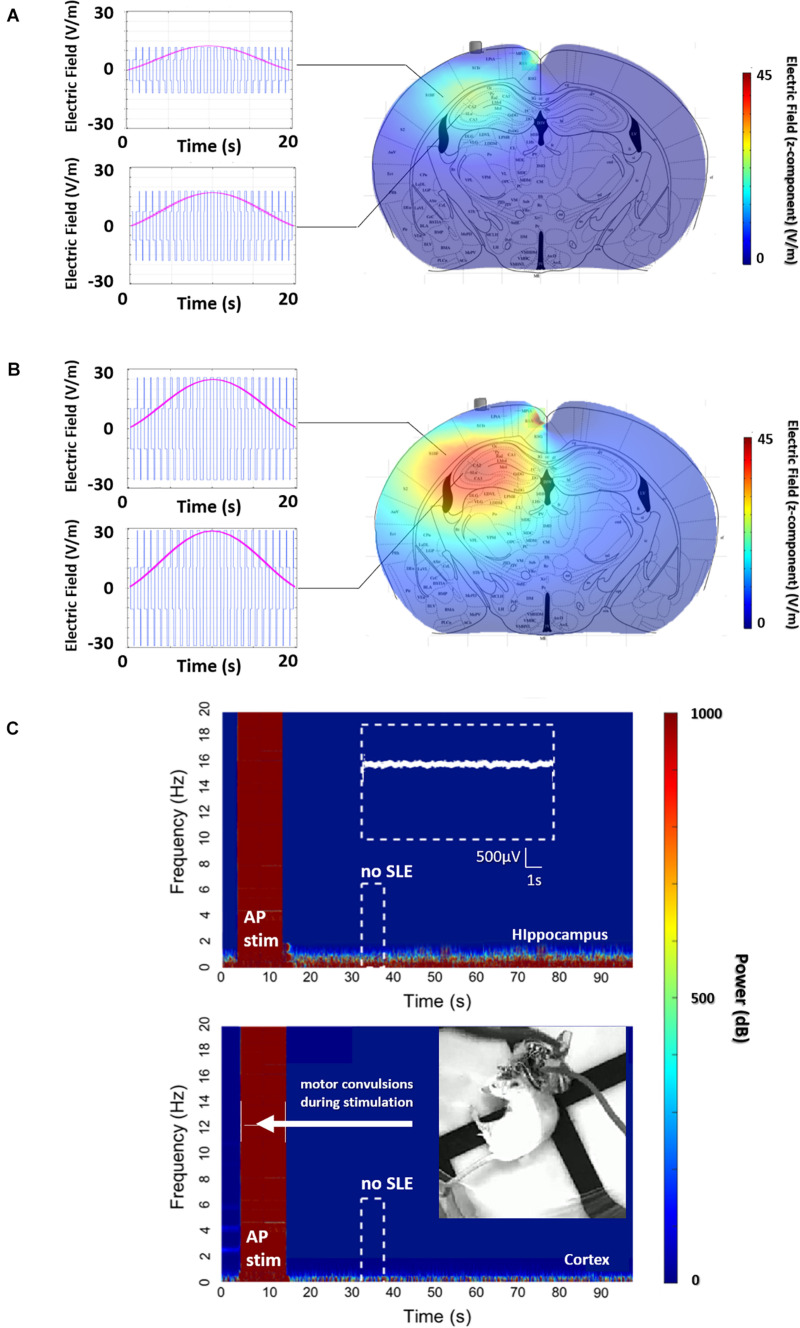
AP, non-preferential orientation, electric field envelope versus anatomy. **(A)** Profile of the electric field and envelope for the stimulation in Figure 2D (600 μA per pair), and **(B)** stimulation with the same orientation but with a higher stimulation amplitude (1500 μA per pair). Clearly, continuously increasing the intensity of stimulation will increase the envelope of stimulation in the hippocampus, ideally leading to an SLE. However, the increase in stimulation amplitude creates a non-trivial increase of the envelope located in the cortex. **(C)** This envelope in the cortex is enough to evoke arbitrary activity and motor convulsions. As the stimulation intensity is increased for the AP orientation, significantly past the threshold for SLE in part B, single-sided motor convulsions are observed during the stimulation itself.

An explanation of stimulation using the AP orientation and its subsequent consequences in the motor cortex can be seen in Figures 3A–C, where the envelope of the electric field is compared to the underlying brain anatomy. In Figure 3A, the stimulation is equal to the stimulation applied in Figure 2 with no evoked SLE, and a maximum envelope of electric field is seen at the CA3-CA1 junction in the hippocampus with a small envelope in the neighboring cortex. In Figure 3B, the stimulation from the electrode pairs is increased and correspondingly the maximum envelope in the hippocampus increases, however, so does the envelope in the neighboring cortex.

This increase in stimulation results in Figure 3C, where a maximum limit of stimulation is reached as single-sided (corresponding to the implanted hemisphere) motor convulsions occur during the stimulation itself.

In summary, in Figure 3A, corresponding to the stimulation applied in Figure 2, the amplitude of the electric field is clearly higher at the focal point of the coordinates in the hippocampus. However, due to the non-preferential orientation of the electric field to the axons of the hippocampus, the threshold to evoke an SLE has not been reached.

As amplitude increases to find the threshold for an SLE, a limitation of the TI method is observed with direct consequences for uncontrolled or indiscriminate orientations of TI electric fields. Namely, as the amplitude of the focal spot increases for non-preferential orientations, so does the envelope along a radial axis moving away from the focal spot. A target structure along the radial path may have a threshold for activation, in this case convulsions in the motor cortex, which will be reached before the threshold for activation of the target, in this case seizure activity in the hippocampus. This understanding can be algorithmically formulated and used to tailor stimulation orientations, not limited to AP/ML, for other subcortical structures.

**FIGURE 4:**
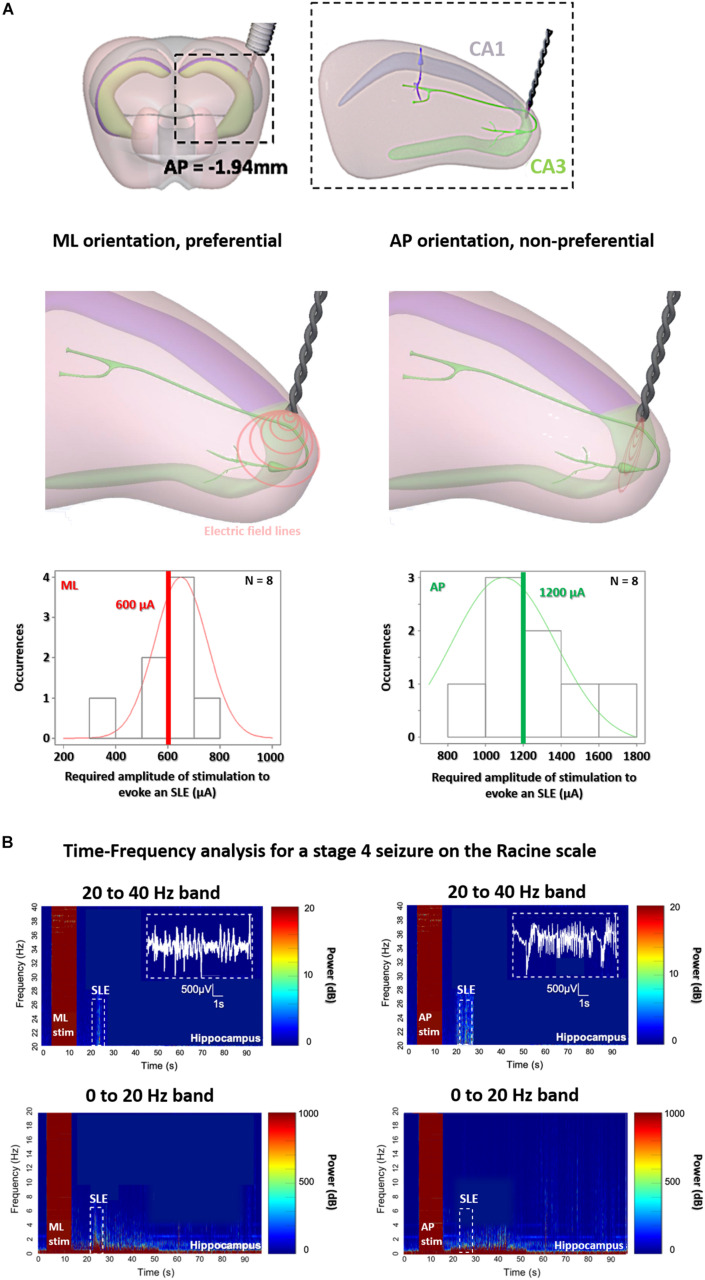
Orientation-controlled stimulation of SLEs in the hippocampus with classic implanted stimulators. **(A)** Stimulation at the border of CA3 and CA1, for implantable electrodes with preferential medio-lateral (ML) orientations having equipotential surfaces of electric field parallel to axons, and with non-preferential anterior-posterior (AP) orientations having equipotential surfaces of electric field perpendicular to axons. **(B)** As soon as the stage 4 on the Racine scale is reached, an increase in power intensity is shown with a peak in beta/gamma range oscillations between 20–40 Hz, correlating with SLEs (15 s after the stimulation) for implantable orientations, however, for AP (right panels) double the current is required.

To support the existence of orientation dependent thresholds in TI, we subsequently demonstrate the dependence of orientation on thresholds using implantable electrodes. As seen in Figure 4A (additionally in Figure 6B), we see that when using classic implanted stimulation electrodes, the threshold to evoke SLEs is highly dependent on the orientation of the electric field. For a preferential ML orientation with the equipotential surfaces of the electric field parallel to axons (where a gradient of potential is created along the axons), the threshold requires half the injected charge. For a non-preferential AP orientation with the equipotential surfaces of the electric field perpendicular to axons (where no gradient of potential is created, axons are fixed at one potential surface), the threshold requires double the injected charge.

**FIGURE 5:**
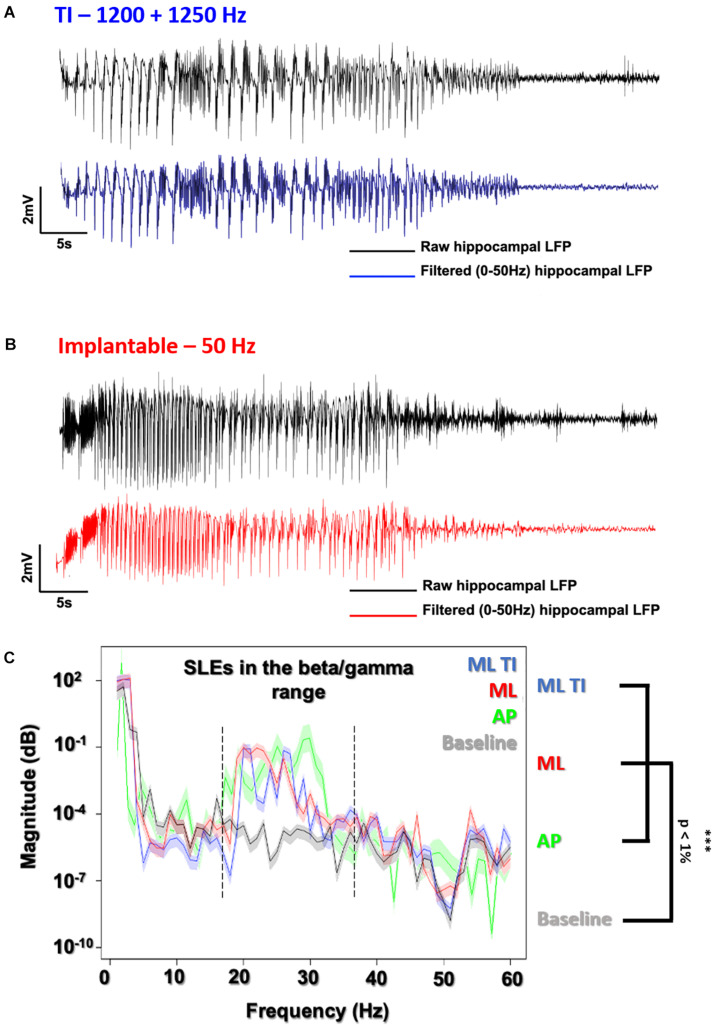
Evoked SLEs using TI vs. implantable electrodes. LFP recording of freely moving mice after a 600 μA stimulation using the preferential ML orientation. **(A)** All recordings (ML-ot-TI in blue and ML-implantable in red) were analyzed using a 50 Hz lowpass filter to clearly distinguish the SLE. **(B)** The classic implantable stimulator provides a 50 Hz applied stimulation in the hippocampus. TI provides a 50 Hz stimulation, corresponding to the differences between the two stimulation frequencies in the hippocampus (1200 and 1250 Hz). **(C)** Power Spectral Density (PSD) shows SLEs (15 s after the stimulation) with a peak in the 20–40 Hz band (Beta/Gamma) characteristic of a rapid discharge for both implantable orientations (ML and AP) and ML-TI stimulation protocols.

**FIGURE 6:**
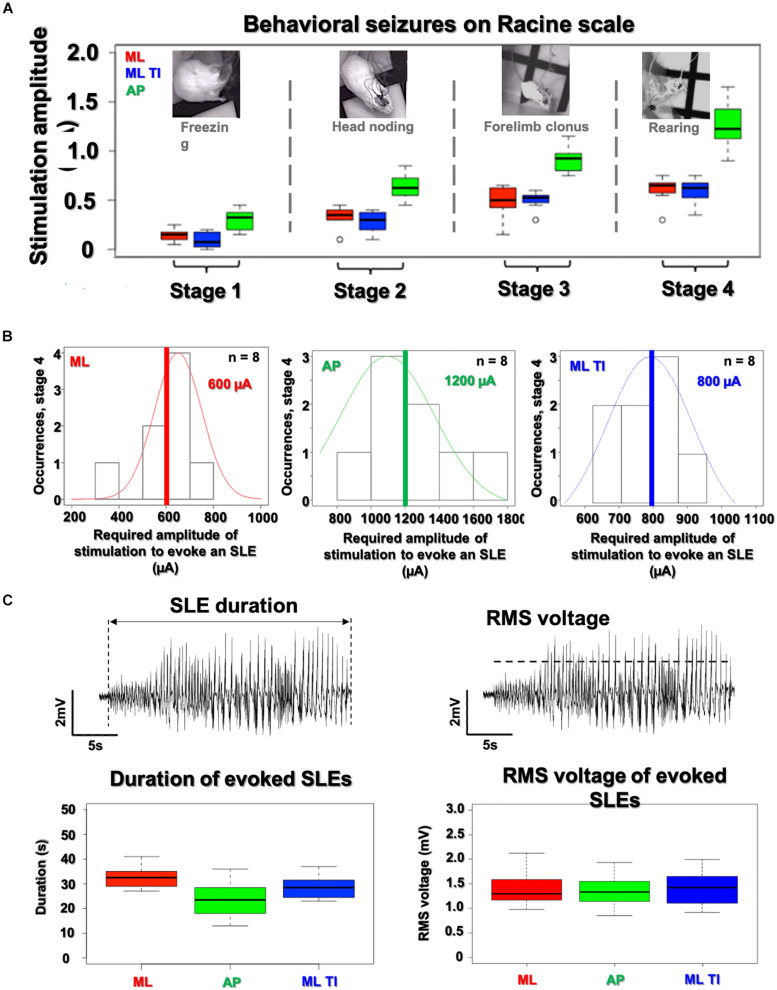
Analysis of SLE characteristics (amplitude, duration, and severity) in the hippocampus. All SLEs detected have been characterize in amplitude, duration and severity. **(A)** No differences are observed between ML-implantable (red) and ML-ot-TI (blue) (*p*-value > 5%) for evoked behavioral changes on the Racine scale as a function of stimulation amplitude. AP requires increased stimulation amplitude for each stage. Black line of box plots represents the median. **(B)** Distribution of SLEs for the 4th stage of the Racine scale, the normalized distribution of thresholds do not show any differences between ML-implantable and ML-ot-TI (*p*-value > 5%), but the AP-implantable stimulation (green) needs double the current to evoke Stage 4 behavior and electrophysiological SLEs. Red, Green, and Blue lines represent the mean. **(C)** The recorded amplitude and duration of evoked electrophysiological SLEs for Racine Stage 4: ML-implantable (red), AP-implantable (green), and ML-ot-TI (blue) show no differences (*p*-value > 5%). Every group of mice in each panels **(A–C)** are *n* = 8.

To be sure that we induced the same SLEs in the two implantable groups, we analyzed and compared the electrophysiology and the behavior at Racine scale 4 in Figure 4B (and additionally in Figure 6A). All mice from the two implantable groups, both ML and AP, exhibit electrophysiological SLEs, with an increase in the beta/gamma band (20–40 Hz), and stage 4 seizures on the Racine scale, however, with a threshold which is significantly lower for the ML orientation. This is an interesting result, as no motor convulsions are observed for the AP orientation with implantable electrodes because there is no axis of stimulation through the cortex, allowing additional insight into the difference in thresholds for preferential and non-preferential orientations of stimulation. Correspondingly, we analyzed and compared the electrophysiology and the behavior for the implantable orientations and ML TI orientation, in Figures 5, 6.

### ML TI and Implantable Stimulation Produce the Same Behavioral and Electrophysiological Results

In Figure 5, electrophysiological SLEs and the associated power spectral density (PSD) are shown, where the PSD is 15 s after the stimulation and seen to have a peak power in beta/gamma range between 20 and 40 Hz, true for implantable orientations and identical for the ML TI.

The three groups with detectable SLEs (AP-implantable, ML-implantable, and ML-ot-TI) are further compared in Figure 6 using scores on the Racine scale for severity, and using the amplitude and duration of the evoked SLEs. In Figure 6A behavioral stages of the Racine scale are shown versus the applied stimulation current. For each phase, it is necessary to apply more current for AP-implantable to reach the behavioral response. This is consistent with the results previously seen in Figure 4 where more current is necessary to evoke an SLE due to the non-preferential orientation of AP. Electrophysiological SLEs are evoked in Stage 4, therefore as seen in Figure 6B, the distributions of stimulation amplitudes to evoke an SLE during Racine stage 4 are shown. The occurrence of a SLEs is seen versus the necessary applied stimulation current. To evoke an SLE, the average current necessary is significantly higher for AP-implantable compared to ML-implantable and ML-ot-TI. Once an occurrence of an SLE has been recorded, the evoked SLEs are specifically compared using amplitude and duration. In Figure 6C, the SLE duration and amplitude distributions for the three groups are shown. Using first a normality test (Shapiro) and then a *t*-test, there were no differences between the three groups for SLEs durations and amplitudes, all are statistically the same with a Wilcoxon-test, and *p*-value > 5%. In summary, although orientation of AP requires more current to evoke behavioral changes and to evoke SLEs, the evoked SLE which is recorded is identical for all three groups. This is very interesting specifically considering the ML orientations, where ML-implantable and ML-TI are statistically identical in term of induced SLEs (amplitudes, durations) and Racine severities, meaning that the minimally invasive method is generating the same electrophysiological and behavioral event, but without the necessity of implanting the tissue.

Indeed, orientation dependence in TI is not surprising, as orientation dependent stimulation is known to exist for implantable electrodes in the field of Parkinson’s disease (Lehto et al., 2017; Reinacher et al., 2017). What is notable, is that identical evoked SLEs in the beta/gamma range can be attained in the hippocampus using temporally interfering electric fields, without the necessity of an implanted stimulator, if care is taken to account for the orientation of the electric field with respect to the axons of the hippocampus. We created both orientations of preferential ML and non-preferential AP mice using ot-TI. Based on our calculation of geometry and field strength, we can locate the focus at the exact intersection of the CA3 and CA1 used in the implanted stimulation experiment.

## Discussion

Clinically, comparing electrical activity from different brain regions, in particular electrically stimulated brain regions, in real-time as seizures emerge is fundamental to the identification of the EZ (Bancaud and Talairach, 1992). Seizure onset is characterized by dramatic changes in brain rhythms with several patterns of onset often observed, namely preictal epileptic spikes, trains of spikes, rapid-discharges, and slow-wave complexes with frequencies involved in the range of beta/gamma (15–30, 20–40 Hz in mesial temporal seizures) (Perucca et al., 2014). Typically, 8–15 implants are located in patient’s brain (generally 0.8 mm diameter, with multiple electrode contacts 2 mm long and 1.5 mm apart), allowing stimulation at various mesial and lateral sites usually using lower frequencies (typically 1–50 Hz, for 5 s) to map functional cortex or to evoke seizure onset (Bartolomei et al., 2017). Unfortunately, there are some parts of the brain which remain unreachable for electrode implant due to the presence of blood vessels, eloquent cortex, or other surgically complicated implant locations and possible subsequent deficits (Catenoix et al., 2009; Säisänen et al., 2010; Rossini et al., 2015). Clearly, there are limitations to the number and placement of intracranial electrodes and, as we have demonstrated here, ot-TI could be an interesting and additional tool for evoking and stimulating seizure onset. The possibility to explore some parts of the brain unreachable by SEEG electrodes could be very interesting during clinical investigation of, for example, the insula as it has a high density of arteries and veins and it is well-known to be a very difficult tissue area of the brain to operate (Uddin et al., 2017). This is important because the insula is also well-known to be implicated in refractory partial epilepsies and can be part of the epileptogenic zone of the patient (Isnard and Mauguière, 2005; Nguyen et al., 2009). Unfortunately, it is commonly described that patients cannot undergo a proper functional mapping of this site because of the risk of hemorrhage during implant (Ryvlin et al., 2006). Correspondingly, TI also has the possibility to reduce the overall number of SEEG electrodes implanted. There are numerous well-documented complications in SEEG electrode surgery, related infections, and complications which can results in necessary explanation of the electrode (Mullin et al., 2016; Bourdillon et al., 2017). A reduction in SEEG electrodes would correspondingly reduce the number of SEEG-related complications.

In summary, TI is a very recently introduced concept for brain stimulation (Grossman et al., 2017; Rampersad et al., 2019; Lee et al., 2020). In the work here, we have utilized a new orientation-tunable form of TI (ot-TI) to evoke SLEs in the mouse hippocampus having the range of beta/gamma frequencies seen in MTLE, but without the necessity of implantable electrodes. Our target was the classic CA3-CA1 border structure used in epilepsy studies where the neurons of the CA and their axons, the Schaffer collaterals, are a well-known target for evoking seizures *in vivo* and *in vitro* with penetrating electrodes (Barbarosie and Avoli, 1997; Pelletier et al., 1999; Schultz and Rolls, 1999). In our alternative technique relying exclusively on non-penetrating surface electrodes and ot-TI electric fields, we have shown that the SLEs generated are indeed focally evoked in the hippocampus and identical to focally evoked SLEs generated with classically implanted electrodes. We additionally demonstrated the use of square-waves with TI stimulation, as clinically, square-waves are most often used in stimulation. As we have shown, the orientation of the electric field plays a key role in evoking events, and if carefully controlled, optimal orientation dramatically lowers thresholds for both implantable electrodes and correspondingly the minimally invasive ot-TI, replicating the implantable stimulation without the need of penetrating the cortex. Currently, non-invasive tools for exploration of brain tissue to identify seizure foci are limited to Transcranial direct current stimulation (tDCS) and Transcranial magnetic stimulation (TMS), both methods limited to a few centimeters in depth (Deng et al., 2013). TI shows promise to be an additional new tool which could significantly advance our capacity of probing the organization of spatiotemporal brain activity and could dramatically increase the explorable tissue for clinical definition of the EZ. One possible technical note is that the electric field at the hippocampal target in our mice with ot-TI was between 20 and 25 V/m which is below the intensity typically presumed necessary to induce direct activation of neurons with TMS (60–80 V/m). If this possible technical note can be addressed in future work, it could additionally make it possible to deliver therapeutic stimulation in a highly controlled and accurate non-invasively stimulation to deeper regions of the brain, an interesting topic for a subsequent study. Regardless, the technique undoubtedly holds great promises with potential applications in epilepsy, but also for a wide range of other brain disorders currently managed by electrical brain stimulation.

## Materials and Methods

### Animals

All experiments were performed in accordance with European Council Directive EU2010/63, and French Ethics approval (Williamson, n. APAFIS 20359 - 2019041816357133). For this study we used 4 groups of 8 male OF1 mice (Charles Rivers Laboratories, France) aged 8–10 weeks. Animals were kept in transparent cages in groups of three to five, in a temperature-controlled room (20 ± 3°C) with a 12/12 h night-day cycle. All animals had *ad libitum* access to food and water. Mice were divided into cohorts, differentiated by the electrode orientation: medio-lateral (ML) and anterior-posterior (AP) for both implanted and ot-TI. Additional mice were assigned for ot-TI studies with additionally implanted depth recording electrodes in the hippocampus to record the dynamics of the evoked SLE in the hippocampus vs. the cortex. After the surgery, all mice were kept in separate cages to avoid fighting and to avoid damage to implanted electrodes.

### Surgical Procedure

The 32 mice were anesthetized via an intraperitoneal injection of ketamine (50 mg/kg) and xylazine (20 mg/kg) and placed in a stereotaxic frame with the head adjusted for bregma and lambda in the same horizontal plane. After midline scalp incisions, the following stereotaxic coordinates were used for craniotomies: [AP: −1.94, ML: +0.5; −0.5; −3.9; −4.3] for the ML electrode orientation and [AP: +2.2; +1.1; −4.4; −5.5; ML: −2.04]. For the implantable twisted-pair platinum electrodes (from PlasticsOne; wire length = 2 mm, individual wire diameter = 125 μm), coordinates were AP: −1.94, ML: 2.8, DV: 1.57 using a 15-degree angle. All the coordinates were calculated using the Paxinos Atlas. *Dura mater* was gently removed and four stainless steel mini-screws of 0.8 mm diameter (Component Supply, Miniature Stainless Steel Self-Tapping Screws: TX00-2FH) were placed on the cortex without penetration into the brain tissue. Subsequently, dental cement (Phymep, SuperBond) was applied on the skull surface to fix the screws and the skull cap was formed using Dentalon. During the post-surgical recovery time (3 days), all the mice were observed for signs of pain, distress and neurological complications.

### TI Electrical Stimulation

Electrical stimulation was performed using electrical stimulators (IntanTech, Intan 128ch Stimulation/Recording Controller) with two frequencies, 1200 and 1250 Hz, and biphasic, bipolar pulses of 500 μs with the overall stimulation time of 10 s (complete details in Supplementary Figures 1, 2). Additional details on isolation of stimulation systems were described in Supplementary Figure 4. The stimulation amplitude was increased in 50 μA steps, starting from 50 μA, until reaching a seizure of stage 4 on the Racine scale for ML or until an animal exhibited an intense motor response (i.e., convulsions in AP orientation). Control stimulations (1200/1200 and 50/50 Hz) were performed at the threshold intensity of the TI session, with no observed SLEs. As expected, with no frequency offset, there is no envelope and thus no stimulation of the hippocampus. Details are shown in Supplementary Figure 9. Each mouse received one full TI stimulation to identify the threshold and two subsequent control stimulations. The investigator characterizing the electrophysiologically evoked response was not-informed of the group.

### Direct Stimulation

After 3 days of recovery following implantation, a protocol to induce and to characterize the AD threshold was applied using the Racine scale. Following previous work, ADs were defined as high-amplitude spikes and polyspike epileptiform events visible after the applied stimulus. We used a 10 s stimulation, bipolar, biphasic, at 50 Hz with a 500 μs pulse width, with the amplitude increased in 50 μA steps, starting from 50 μA, until reaching a seizure of stage 4 on the Racine scale. The animal was given 30 min rest between attempted stimulations. The investigator characterizing the electrophysiologically evoked response was not-informed of the group.

### Behavioral Evaluation

All mice were video monitored during stimulation. Behavioral responses were analyzed, taking care to distinguish between a motor response and an epileptic seizure. For seizure detection the following criteria were used, according to the Racine scale: Racine scale: Stage 0, no visible change in behavior; Stage 1, freezing with facial movements; Stage 2, head nodding; Stage 3, forelimb clonus; Stage 4, rearing without loss of balance; and Stage 5, rearing and loss of balance). The motor cortex response was evaluated, comparing the video recording with the simultaneous EEG recording, depth electrodes, and by extracting non-specific signs (limb twitching, vocalizing, etc.). In parallel to the LFP recording, video monitoring was continued during the complete stimulation/recording/rest sessions of the freely moving animal in its cylinder open-field environment (50 cm × 25 cm × 25 cm) to monitor the behavior and correlate it directly with the brain activity.

### Virtual Simulation

COMSOL Multiphysics, version 5.5^1^ was used to create Finite-element method simulations of the electrodes and environment. Prior to simulations, we created using Hexagon software a fine 3D mouse brain based on the Paxinos mouse brain atlas. Detailed information regarding the mesh parameters for the FEM are in Supplementary Figure 5.

We embedded the stimulation electrodes (stimulation and grounds) as platinum stimulators with the tissue model environment having the frequency-dependent complex permittivity values of brain tissue. Using electrical stimulation from our electrodes in COMSOL solving the Maxwell equations with our mouse brain mesh, the equipotential surfaces and electric field lines applied by the electrodes could be visualized in the hippocampus. The orientation and positioning of the electrodes could then be modified to better target the border of the CA3 with the maximum of the envelope located at the CA3 border (matching exactly the stereotactic targets show in the surgery section). The physical extent of field lines with respect to the orientation of the axons of pyramidal cells in the hippocampus could be better seen.

### Statistical Analysis

All raw recordings were plotted and analyzed via Matlab using raw data or post-processed data using Hilbert function to calculate stimulation envelops. Statistical analyzes (R software) of AD-thresholds were performed using the parametric one-way ANOVA and pairwise *T*-tests to calculate the probably significant differences in SLE amplitude, duration or severity between the 4 groups. For the TI group, the AD-threshold distribution appears to not follow a normal distribution. Therefore, all AD-thresholds were analyzed using a standard non-parametric Wilcoxon-Mann-Whitney tests. An ANOVA was also performed to analyzed the PSD of each stimulation groups that could elicit a stage 4 seizure on Racine scale. For all groups, the ANOVA didn’t show any differences in the groups themselves but a global differences between baseline vs. stimulation groups was demonstrated. SLE duration was the total duration of the electrographic spike activity (amplitude > 2 × baseline), with the signal high-pass filtered at 1 Hz. SLE amplitude was calculated by taking the RMS of the voltage inside the SLE duration window.

### Cluster Analysis for SLE Detection

Cluster analysis for SLE detection (Supplementary Figures 8, 9): recordings from each group (TI, implanted, baseline, controls), *n* = 8 recordings for each group, were rms-averaged to create an averaged recording. The averaged recordings were cluster analyzed to visualize *inter*-group differences. Each averaged recording was clustered in 50 ms windows. The cluster analysis consists of comparing a cluster from one group to all other clusters from a different group using *t*-tests to visualize the results as a matrix of *t*-values (Supplementary Figures 8, 9). This shows seizure onset in the stimulated groups as a difference in *t*-value for TI and implanted vs. baseline, and shows no differences in *t*-value for TI vs. implanted (because the evoked seizures are the same). As seen, the control groups (for example stimulation with only 1200 Hz) shows no difference from baseline, as no activity was evoked in the hippocampus. Additional cluster analysis was performed to visualize *intra*-group differences, results not shown as cluster analysis within a single group yields similar results (for example, “baseline” is also present at the beginning and end of recordings with seizures, and similar matrix is seen). The detected SLE using *t*-values gives similar onset time and duration as the method of duration used above, by calculating duration of electrographic spike activity (amplitude > 2 × baseline).

## Data Availability Statement

The raw data supporting the conclusions of this article will be made available by the authors, without undue reservation.

## Ethics Statement

The animal study was reviewed and approved by French Ethics: Comité d’éthique en expérimentation animale n°071, (Williamson, n. APAFIS 20359 – 2019041816357133).

## Author Contributions

AW conceived the project. ER, EA, and FM performed the experiments. BB performed the finite-element simulations. FM analyzed the neural data. AW wrote the manuscript with input from the other authors, including RC, VJ, AT, and FB. All authors contributed to the article and approved the submitted version.

## Conflict of Interest

The authors declare that the research was conducted in the absence of any commercial or financial relationships that could be construed as a potential conflict of interest.

## Footnotes

1 www.comsol.com

## Supplementary Material

Supplementary Figure 1 Square-wave Ti. Using square waves for Ti is the same as using sine waves. Practically, this does not appear as an envelope on the screen during stimulation, rather it appears as a “synchronization rate” – essentially the two square pulses coming closer and closer into phase. However, when the signal is filtered, one will visualize the low frequency envelope as expected. The envelope is calculated by taking the Hilbert transform (orange) of the recorded signal.

Supplementary Figure 2 Stimulation Protocol for Ti and implantable. Each session consists of the 10 s stimulation and 5 min Eeg/video monitoring, during which animal’s behavior is observed and analyzed, in accordance with simultaneous Eeg-recording. Once an Sle is detected, the stimulation threshold is noted, no further stimulation is necessary.

Supplementary Figure 3 Field lines of Ti. The field lines in Figure 1 of the main manuscript are plotted to highlight the symmetry of the Ti field with respect to the orientation of axons in the Ca of the hippocampus. Complete plots of the field lines, as show here for the Ml orientation, also include lines at angles deviating from the primary symmetry. However, as pictured in Figure 2 of the main manuscript, the field lines along the axis of symmetry have the largest relevant envelope due to the orientation of the two pairs of electrodes.

Supplementary Figure 4 How Not to make a Ti field. It is extremely important that the two stimulation systems are isolated. If the stimulation systems share the same reference/ground the situation pictured in the figure here will occur. Field lines from the cathode of one stimulation pair will travel to the anode of the opposite stimulation pair. This will significantly weaken, and possibly completely remove, the point of the maximum envelope of stimulation (red). This can often not simply be done by connecting two separate stimulators, as both machines will be connected to the same wall socket. Ideally two separate battery-powered stimulators will provide the perfect isolation.

Supplementary Figure 5 Finite-element model, meshing. The figure contains the meshing parameters used for the Fem simulations of the mouse brain. Both the brain-outline and subcortical structures are taken from the Atlas, the Ca3 of the hippocampus is highlighted in the figure. Complex permittivity as a function of frequency, values taken from literature, was used for epsilon when solving the Maxwell equations. **(A)** Mouse brain structures are highlighted in the Allen Atlas, and the subsequent 3D structure is extracted from the Atlas. **(B)** The 3D structure is imported into Comsol and an extra fine mesh is used. Stimulation electrodes were then added to cortical locations and the amplitude of the electric field, in our case the maximum of the envelope, could be visualized. Electrode placements can then be easily shifted to tailor the location of the maximum of the envelope to the structure of interest, in our case the border of the Ca3.

Supplementary Figure 6 Placement of recording electrodes in cortex and hippocampus. A recording electrode is implanted in the hippocampus and Ti stimulation electrodes on the cortex are used as cortical recording electrodes after stimulation. The location of the hippocampal recording electrode can be seen in panel 3, placed in the Ca1.

Supplementary Figure 7 Time Frequency of cortex in the 20 Hz to 40 Hz. No perturbation resulting from the stimulation are seen in the frequency region in the cortex due to stimulation in the hippocampus using both Ti or implanted electrodes.

Supplementary Figure 8 Cluster-based analysis of Sle beginning. A cluster-based analysis (cluster size = 20 ms) has been conducted to ensure that the fluctuations seen in both the recorded raw signal and the Rms signal were due to Sles beginning, and not due to random activity. **(A)** All detected Sles are Rms averaged and cluster analyzed to view the Sle beginning and end visually with a *t*-score. **(B,C)** Onset of Sles induce a significant difference in recordings compared to the baseline recorded in the hippocampus as seen by *t*-score. **(D)** Moreover, when comparing Ml Ti induced Sles and Ml induced Sles, no significant differences are observed using the cluster permutation technique. The results are consistent with the Psd shown in Figure 5, where Ml Ti and Ml implantable induced equivalent beta/gamma oscillations and Sles compared to the baseline recorded. Additionally, control stimulations using only the envelope frequency applied to the cortex show no significant difference to baseline using *t*-score (not shown).

Supplementary Figure 9 Control experiments for Ti stimulation. **(A)** there is no difference in electrophysiological activity recorded in the hippocampus between baseline data, cortical stimulation at 50 Hz, or cortical stimulation at 1200 Hz (no envelope). Each group is *n* = 4. **(B)** Similar cluster-based analysis as seen in Supplementary Figure 8 (cluster size = 20 ms). The *t*-score for recordings in the hippocampus during low-frequency stimulation provided to the cortex compared to baseline recordings in the hippocampus show no significance.

## References

[B1] Alarcon G. Binnie C. D. Elwes R. D. C. Polkey C. E. (1995). Power spectrum and intracranial Eeg patterns at seizure onset in partial epilepsy. *Electroencephalogr. Clin. Neurophysiol.* 94 326–337. 10.1016/0013-4694(94)00286-T 7774519

[B2] Allen P. J. Fish D. R. Smith S. J. M. (1992). Very high-frequency rhythmic activity during SEEG suppression in frontal lobe epilepsy. *Electroencephalogr. Clin. Neurophysiol.* 82 155–159. 10.1016/0013-4694(92)90160-J 1370786

[B3] Bancaud J. Talairach J. (1992). Clinical semiology of frontal lobe seizures. *Adv. Neurol.* 57 3–58. 1543059

[B4] Barbarosie M. Avoli M. (1997). CA3-Driven Hippocampal-Entorhinal Loop Controls Rather than Sustains *In Vitro* Limbic Seizures. *J. Neurosci.* 17 9308–9314. 10.1523/JNEUROSCI.17-23-09308.1997 9364076 PMC6573610

[B5] Bartolomei F. Chauvel P. Wendling F. (2008). Epileptogenicity of brain structures in human temporal lobe epilepsy: a quantified study from intracerebral EEG. *Brain* 131 1818–1830. 10.1093/brain/awn111 18556663

[B6] Bartolomei F. Lagarde S. Wendling F. McGonigal A. Jirsa V. Guye M. (2017). Defining epileptogenic networks: Contribution of SEEG and signal analysis. *Epilepsia* 58 1131–1147. 10.1111/epi.13791 28543030

[B7] Bartolomei F. Wendling F. Bellanger J.-J. Régis J. Chauvel P. (2001). Neural networks involving the medial temporal structures in temporal lobe epilepsy. *Clin. Neurophysiol.* 112 1746–1760. 10.1016/S1388-2457(01)00591-0 11514258

[B8] Bourdillon P. Ryvlin P. Isnard J. Montavont A. Catenoix H. Mauguière F. (2017). Stereo-electroencephalography (SEEG) is a safe procedure, including for insular implantations. *World Neurosurg.* 99 353–361. 28003163 10.1016/j.wneu.2016.12.025

[B9] Catenoix H. Guénot M. Mauguière F. Isnard J. (2009). Thermocoagulations multiples guidées par la SEEG et malformations de développement cortical. *Epilepsies* 21:9.

[B10] Cela E. McFarlan A. R. Chung A. J. Wang T. Chierzi S. Murai K. K. (2019). An Optogenetic Kindling Model of Neocortical Epilepsy. *Sci. Rep.* 9:5236. 10.1038/s41598-019-41533-2 30918286 PMC6437216

[B11] Deng Z.-D. Lisanby S. H. Peterchev A. V. (2013). Electric field depth–focality tradeoff in transcranial magnetic stimulation: Simulation comparison of 50 coil designs. *Brain Stimulat.* 6 1–13. 10.1016/j.brs.2012.02.005 22483681 PMC3568257

[B12] Frauscher B. Bartolomei F. Kobayashi K. Cimbalnik J. van ‘t Klooster M. A. Rampp S. (2017). High-frequency oscillations: The state of clinical research. *Epilepsia* 58 1316–1329. 10.1111/epi.13829 28666056 PMC5806699

[B13] Grossman N. Bono D. Dedic N. Kodandaramaiah S. B. Rudenko A. Suk H.-J. (2017). Noninvasive Deep Brain Stimulation via Temporally Interfering Electric Fields. *Cell* 169 1029.e–1041.e. 10.1016/j.cell.2017.05.024 28575667 PMC5520675

[B14] Guye M. Régis J. Tamura M. Wendling F. McGonigal A. Chauvel P. (2006). The role of corticothalamic coupling in human temporal lobe epilepsy. *Brain* 129 1917–1928. 10.1093/brain/awl151 16760199

[B15] Hutcheon B. Yarom Y. (2000). Resonance, oscillation and the intrinsic frequency preferences of neurons. *Trends Neurosci.* 23 216–222. 10782127 10.1016/s0166-2236(00)01547-2

[B16] Isnard J. Mauguière F. (2005). Le lobe de l’insula et les épilepsies partielles. *Revue Neurol.* 161 17–26. 10.1016/S0035-3787(05)84970-7 15677998

[B17] Kabakov A. Y. Muller P. A. Pascual-Leone A. Jensen F. E. Rotenberg A. (2012). Contribution of axonal orientation to pathway-dependent modulation of excitatory transmission by direct current stimulation in isolated rat hippocampus. *J. Neurophysiol.* 107 1881–1889. 10.1152/jn.00715.2011 22219028 PMC3331663

[B18] Koessler L. Benar C. Maillard L. Badier J.-M. Vignal J. P. Bartolomei F. (2010). Source localization of ictal epileptic activity investigated by high resolution EEG and validated by SEEG. *Neuroimage* 51 642–653. 20206700 10.1016/j.neuroimage.2010.02.067

[B19] Lagarde S. Bonini F. McGonigal A. Chauvel P. Gavaret M. Scavarda D. (2016). Seizure-onset patterns in focal cortical dysplasia and neurodevelopmental tumors: Relationship with surgical prognosis and neuropathologic subtypes. *Epilepsia* 57 1426–1435. 10.1111/epi.13464 27406939

[B20] Lee S. Lee C. Park J. Im C.-H. (2020). Individually customized transcranial temporal interference stimulation for focused modulation of deep brain structures: a simulation study with different head models. *Sci. Rep.* 10:11730. 10.1038/s41598-020-68660-5 32678264 PMC7366675

[B21] Lehto L. J. Slopsema J. P. Johnson M. D. Shatillo A. Teplitzky B. A. Utecht L. (2017). Orientation selective deep brain stimulation. *J. Neural. Eng.* 14:016016. 10.1088/1741-2552/aa5238 PMC5440185 28068296

[B22] Lian J. Bikson M. Sciortino C. Stacey W. C. Durand D. M. (2003). Local Suppression of Epileptiform Activity by Electrical Stimulation in Rat Hippocampus *In Vitro*. *J. Physiol.* 547 427–434. 10.1113/jphysiol.2002.033209 12562909 PMC2342650

[B23] Mirzakhalili E. Barra B. Capogrosso M. Lempka S. F. (2020). Biophysics of Temporal Interference Stimulation. *Cell Syst.* 11 557.e–572.e. 33157010 10.1016/j.cels.2020.10.004

[B24] Mullin J. P. Shriver M. Alomar S. Najm I. Bulacio J. Chauvel P. (2016). Is SEEG safe? A systematic review and meta-analysis of stereo-electroencephalography-related complications. *Epilepsia* 57 386–401. 10.1111/epi.13298 26899389

[B25] Munari C. Kahane P. Tassi L. Francione S. Hoffmann D. Russo G. L. (1993). “Intracerebral Low Frequency Electrical Stimulation: a New Tool for the Definition of the ‘Epileptogenic Area’?,” in *Advances in Stereotactic and Functional Neurosurgery 10*, eds Meyerson B. A. Broggi G. Martin-Rodriguez J. Ostertag C. Sindou M. (Vienna: Springer), 181–185. 10.1007/978-3-7091-9297-9_42 8109287

[B26] Musto A. E. Samii M. S. Hayes J. F. (2009). Different phases of afterdischarge during rapid kindling procedure in mice. *Epilepsy Res.* 85 199–205. 10.1016/j.eplepsyres.2009.02.020 19375287 PMC2713808

[B27] Nguyen D. K. Nguyen D. B. Malak R. Leroux J.-M. Carmant L. Saint-Hilaire J.-M. (2009). Revisiting the role of the insula in refractory partial epilepsy. *Epilepsia* 50 510–520. 10.1111/j.1528-1167.2008.01758.x 18717706

[B28] Oderiz C. C. Gotman J. Hall J. Hincapié A.-S. Hoffmann D. Job A.-S. (2019). Association of Cortical Stimulation–Induced Seizure With Surgical Outcome in Patients With Focal Drug-Resistant Epilepsy. *JAMA Neurol.* 76 1070–1078. 10.1001/jamaneurol.2019.1464 31180505 PMC6563597

[B29] Pelletier M. R. Wadia J. S. Mills L. R. Carlen P. L. (1999). Seizure-Induced Cell Death Produced by Repeated Tetanic Stimulation In Vitro: Possible Role of Endoplasmic Reticulum Calcium Stores. *J. Neurophysiol.* 81 3054–3064. 10.1152/jn.1999.81.6.3054 10368420

[B30] Perucca P. Dubeau F. Gotman J. (2014). Intracranial electroencephalographic seizure-onset patterns: effect of underlying pathology. *Brain* 137 183–196. 10.1093/brain/awt299 24176980

[B31] Rampersad S. Roig-Solvas B. Yarossi M. Kulkarni P. P. Santarnecchi E. Brooks D. H. (2019). Prospects for transcranial temporal interference stimulation in humans: a computational study. *Neuroimage* 202:116124. 10.1016/j.neuroimage.2019.116124 PMC6819277 31473351

[B32] Reinacher P. C. Krüger M. T. Coenen V. A. Shah M. Roelz R. Jenkner C. (2017). Determining the Orientation of Directional Deep Brain Stimulation Electrodes Using 3D Rotational Fluoroscopy. *AJNR Am. J. Neuroradiol.* 38 1111–1116. 10.3174/ajnr.A5153 28385887 PMC7960073

[B33] Rossini P. M. Burke D. Chen R. Cohen L. G. Daskalakis Z. Di Iorio R. (2015). Non-invasive electrical and magnetic stimulation of the brain, spinal cord, roots and peripheral nerves: Basic principles and procedures for routine clinical and research application. An updated report from an I.F.C.N. Committee. *Clin. Neurophysiol.* 126 1071–1107. 10.1016/j.clinph.2015.02.001 25797650 PMC6350257

[B34] Ryvlin P. Minotti L. Demarquay G. Hirsch E. Arzimanoglou A. Hoffman D. (2006). Nocturnal Hypermotor Seizures, Suggesting Frontal Lobe Epilepsy, Can Originate in the Insula. *Epilepsia* 47 755–765. 10.1111/j.1528-1167.2006.00510.x 16650142

[B35] Säisänen L. Könönen M. Julkunen P. Määttä S. Vanninen R. Immonen A. (2010). Non-invasive preoperative localization of primary motor cortex in epilepsy surgery by navigated transcranial magnetic stimulation. *Epilepsy Res.* 92 134–144. 10.1016/j.eplepsyres.2010.08.013 20863666

[B36] Schrader L. M. Stern J. M. Koski L. Nuwer M. R. Engel J. (2004). Seizure incidence during single- and paired-pulse transcranial magnetic stimulation (TMS) in individuals with epilepsy. *Clin. Neurophysiol.* 115 2728–2737. 10.1016/j.clinph.2004.06.018 15546781

[B37] Schultz S. R. Rolls E. T. (1999). Analysis of Information Transmission in the Schaffer Collaterals. *Hippocampus* 9 582–598. 10560929 10.1002/(SICI)1098-1063(1999)9:5<582::AID-HIPO12>3.0.CO;2-P

[B38] Uddin L. Q. Nomi J. S. Hébert-Seropian B. Ghaziri J. Boucher O. (2017). Structure and Function of the Human Insula. *J. Clin. Neurophysiol.* 34 300–306. 10.1097/WNP.0000000000000377 28644199 PMC6032992

[B39] Vitikainen A.-M. Lioumis P. Paetau R. Salli E. Komssi S. Metsähonkala L. (2009). Combined use of non-invasive techniques for improved functional localization for a selected group of epilepsy surgery candidates. *NeuroImage* 45 342–348. 10.1016/j.neuroimage.2008.12.026 19159694

[B40] Wendling F. Bartolomei F. Bellanger J. J. Bourien J. Chauvel P. (2003). Epileptic fast intracerebral EEG activity: evidence for spatial decorrelation at seizure onset. *Brain* 126 1449–1459. 10.1093/brain/awg144 12764064 PMC2040489

