# Supplementary material for: Orientation of Temporal Interference for Non-invasive Deep Brain Stimulation in Epilepsy
Source: Front Neurosci. 2021 Jun 7;15:633988. doi: 10.3389/fnins.2021.633988 (PMC8216218; doi:10.3389/fnins.2021.633988)
Supplement: Supplementary Figure 1 — Square-wave Ti. Using square waves for Ti is the same as using sine waves. Practically, this does not appear as an envelope on the screen during stimulation, rather it appears as a “synchronization rate” – essentially the two square pulses coming closer and closer into phase. However, when the signal is filtered, one will visualize the low frequency envelope as expected. The envelope is calculated by taking the Hilbert transform (orange) of the recorded signal. [file Data_Sheet_1.pdf]

**SUPPLEMENTARY FIGURES**

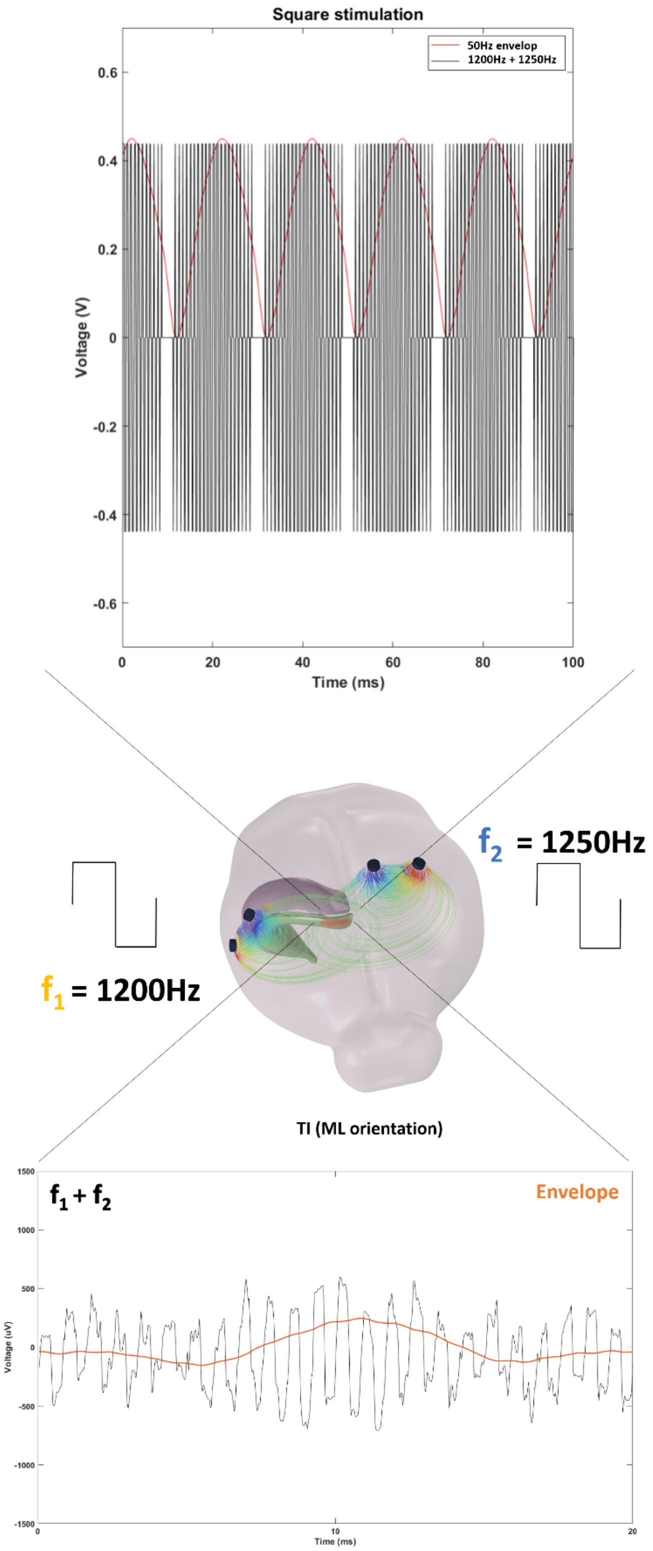

**SUPPLEMENTARY FIGURE S1 | Square-wave TI**. Using square waves for TI is the same as using sine waves. Practically, this does not appear as an envelope on the screen during stimulation, rather it appears as a "synchronization rate" – essentially the two square pulses coming closer and closer into phase. However, when the signal is filtered, one will visualize the low frequency envelope as expected. The envelope is calculated by taking the Hilbert transform (orange) of the recorded signal.

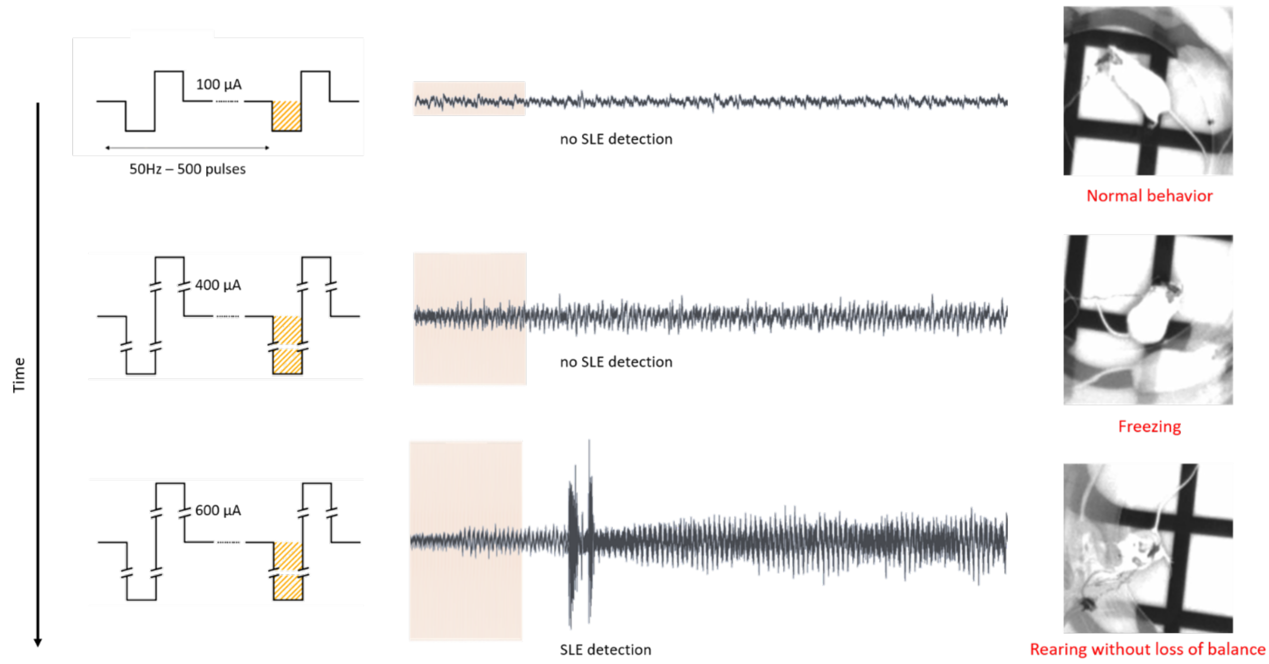


**SUPPLEMENTARY FIGURE S2 | Stimulation Protocol for TI and implantable**. Each session consists of the 10s stimulation and 5min EEG/video monitoring, during which animal's behavior is observed and analyzed, in accordance with simultaneous EEG-recording. Once an SLE is detected, the stimulation threshold is noted, no further stimulation is necessary.

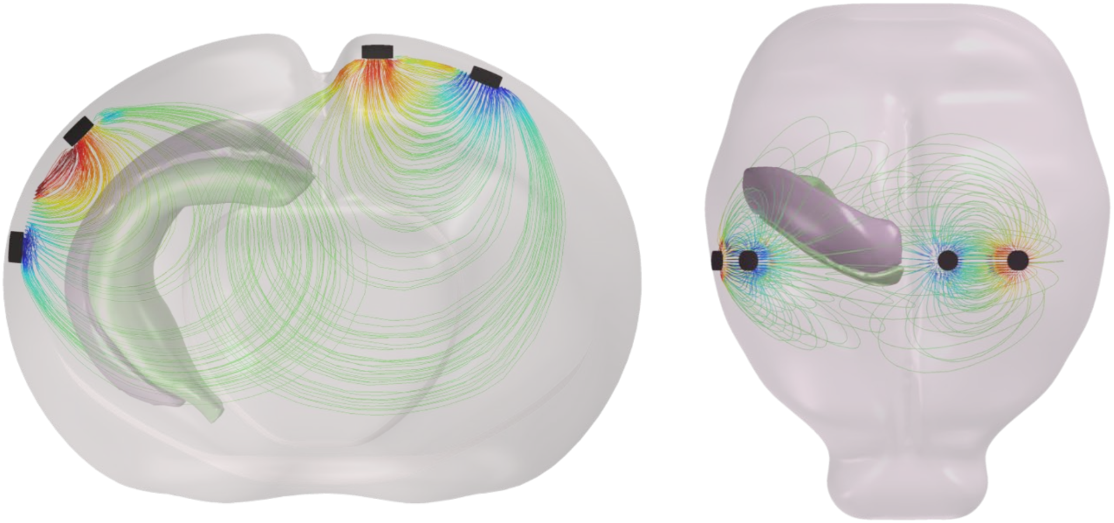

**SUPPLEMENTARY FIGURE S3 | Field lines of TI**. The field lines in **Figure 1** of the main manuscript are plotted to highlight the symmetry of the TI field with respect to the orientation of axons in the CA of the hippocampus. Complete plots of the field lines, as show here for the ML orientation, also include lines at angles deviating from the primary symmetry. However, as pictured in **Figure 2** of the main manuscript, the field lines along the axis of symmetry have the largest relevant envelope due to the orientation of the two pairs of electrodes.

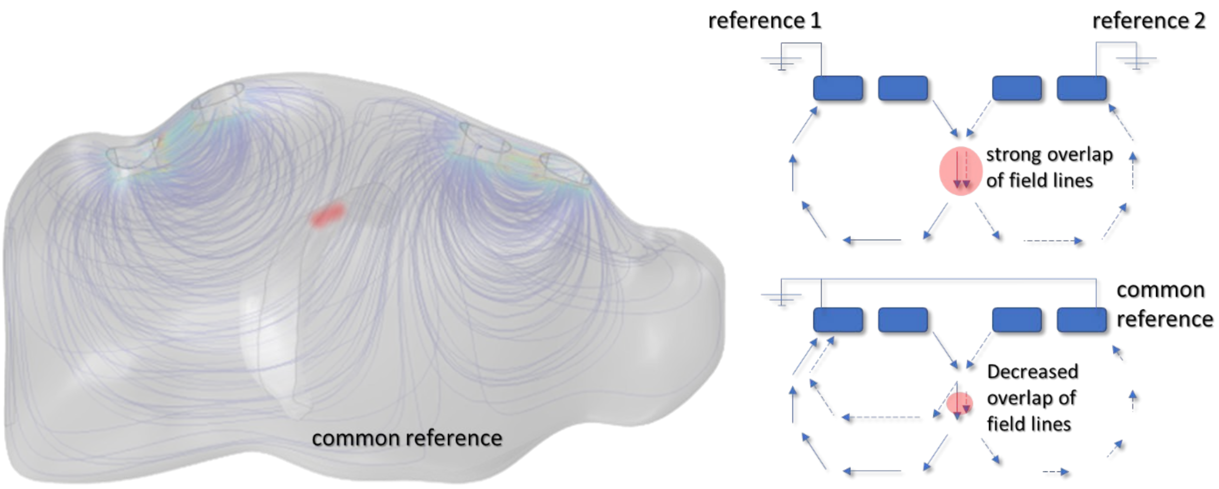


**SUPPLEMENTARY FIGURE S4 | How NOT to make a TI field**. It is extremely important that the two stimulation systems are isolated. If the stimulation systems share the same reference/ground the situation pictured in the figure here will occur. Field lines from the cathode of one stimulation pair will travel to the anode of the opposite stimulation pair. This will significantly weaken, and possibly completely remove, the point of the maximum envelope of stimulation (red). This can often not simply be done by connecting two separate stimulators, as both machines will be connected to the same wall socket. Ideally two separate battery-powered stimulators will provide the perfect isolation.

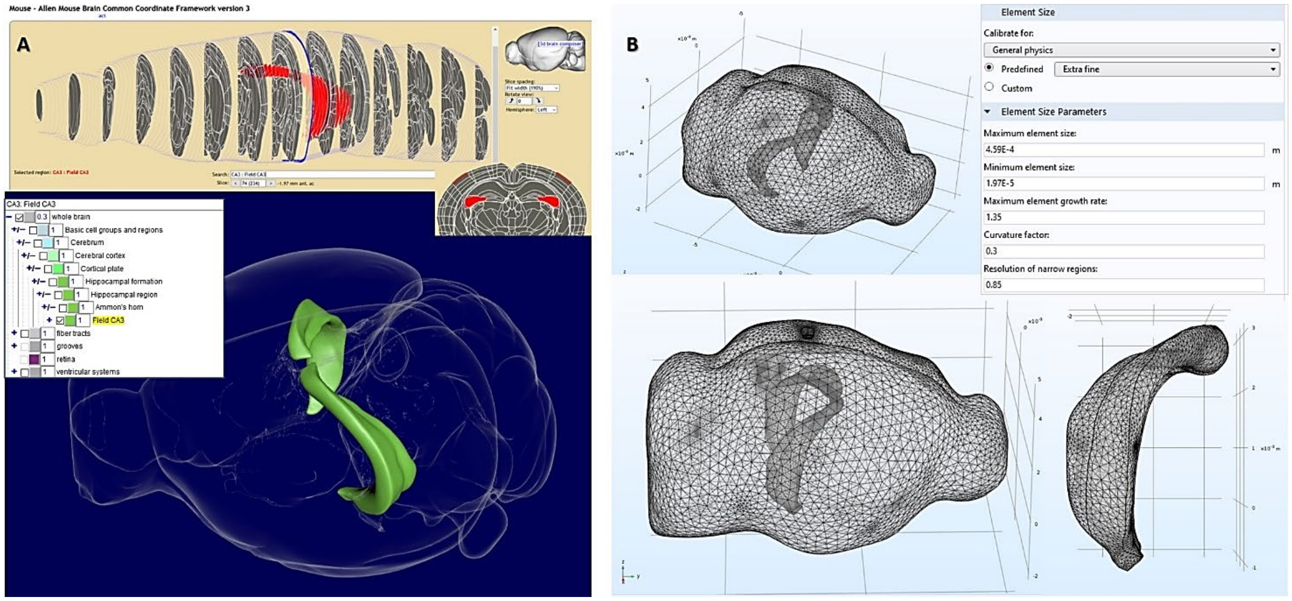


**SUPPLEMENTARY FIGURE S5 | Finite-element model, meshing**. The figure contains the meshing parameters used for the FEM simulations of the mouse brain. Both the brain-outline and subcortical structures are taken from the Atlas, the CA3 of the hippocampus is highlighted in the figure. Complex permittivity as a function of frequency, values taken from literature, was used for epsilon when solving the Maxwell equations. **(A)** Mouse brain structures are highlighted in the Allen Atlas, and the subsequent 3D structure is extracted from the Atlas. **(B)** The 3D structure is imported into COMSOL and an extra fine mesh is used. Stimulation electrodes were then added to cortical locations and the amplitude of the electric field, in our case the maximum of the envelope, could be visualized. Electrode placements can then be easily shifted to tailor the location of the maximum of the envelope to the structure of interest, in our case the border of the CA3.

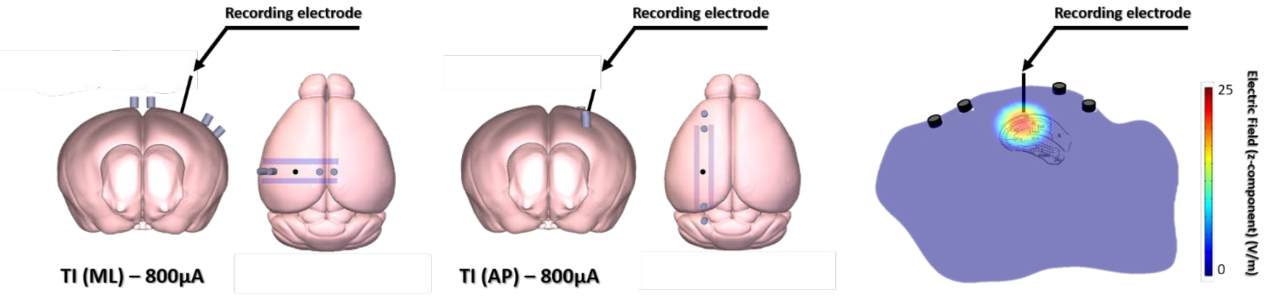


**SUPPLEMENTARY FIGURE S6 | Placement of recording electrodes in cortex and hippocampus.** A recording electrode is implanted in the hippocampus and TI stimulation electrodes on the cortex are used as cortical recording electrodes after stimulation. The location of the hippocampal recording electrode can be seen in panel 3, placed in the CA1.

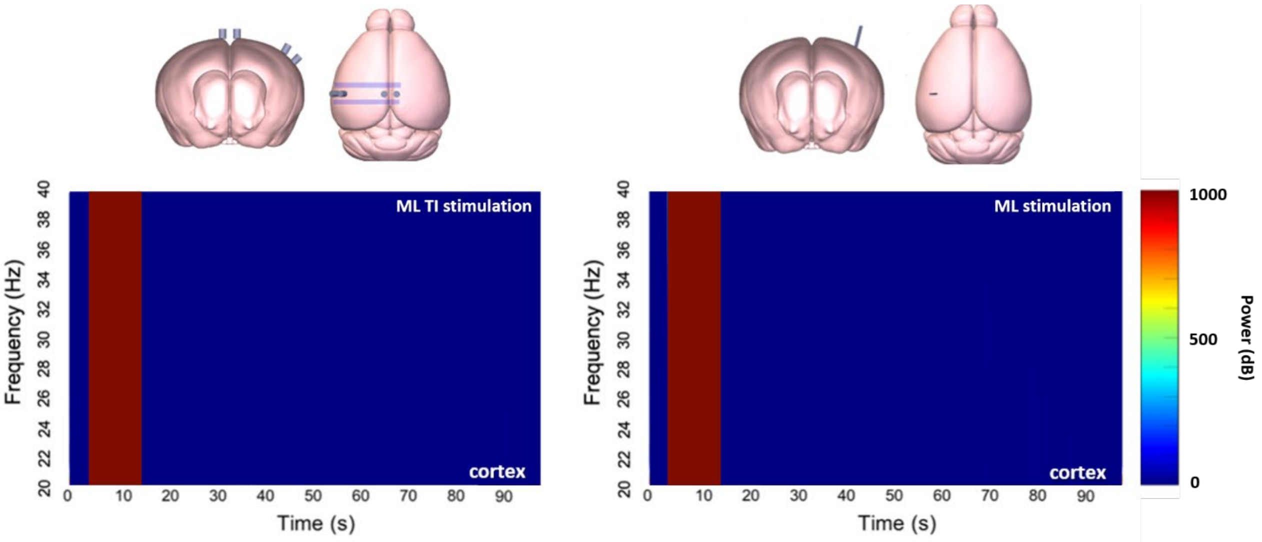


**SUPPLEMENTARY FIGURE S7 | Time Frequency of cortex in the 20 Hz to 40 Hz.** No perturbation resulting from the stimulation are seen in the frequency region in the cortex due to stimulation in the hippocampus using both TI or implanted electrodes.

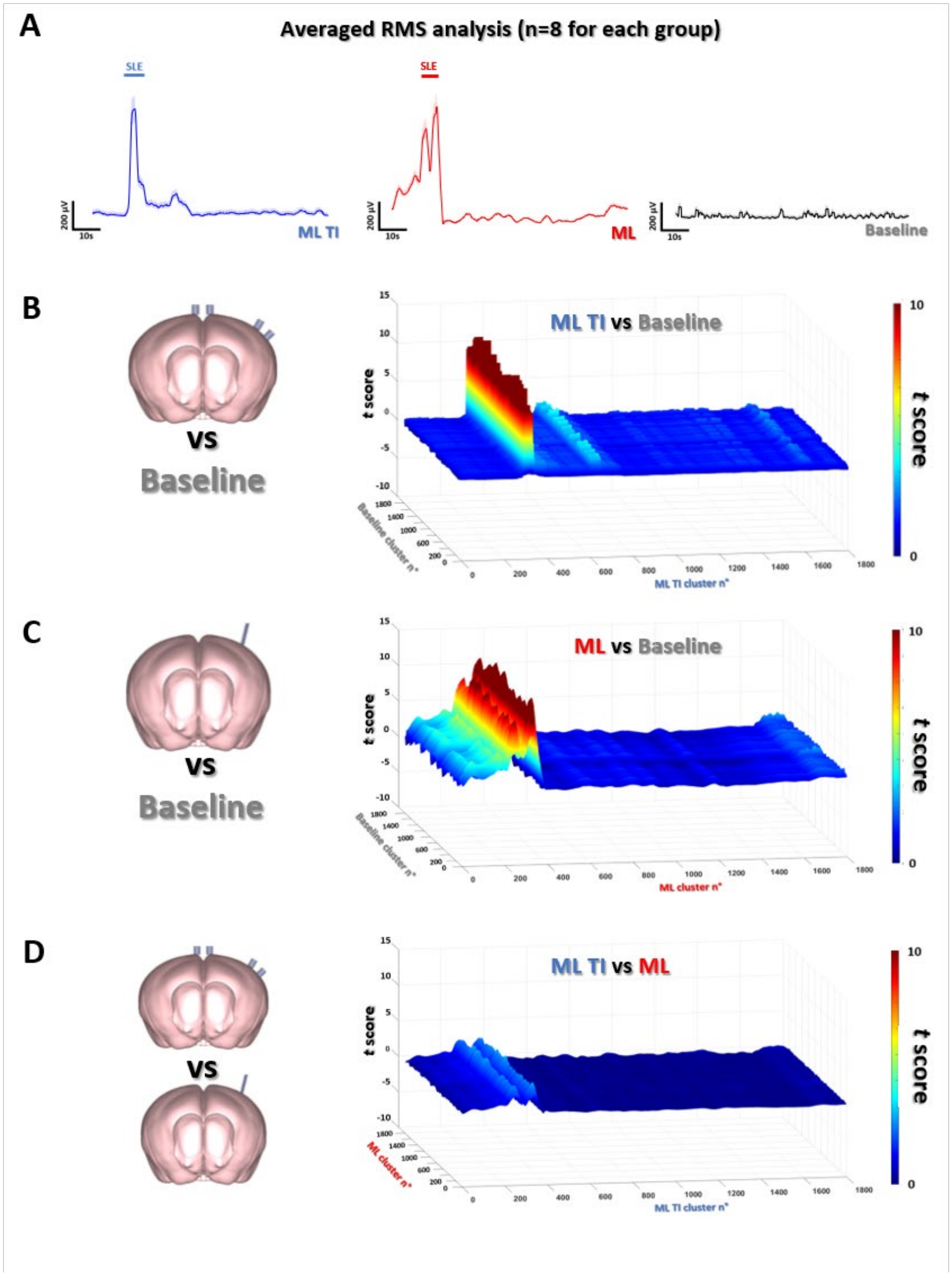


**SUPPLEMENTARY FIGURE S8 | Cluster-based analysis of SLE beginning**. A cluster-based analysis (cluster size = 20ms) has been conducted to ensure that the fluctuations seen in both the recorded raw signal and the RMS signal were due to SLEs beginning, and not due to random activity. **(A)** All detected SLEs are RMS averaged and cluster analyzed to view the SLE beginning and end visually with a t-score. **(B)-(C)** In B and C, onset of SLEs induce a significant difference in recordings compared to the baseline recorded in the hippocampus as seen by t-score. **(D)** Moreover, when comparing ML TI induced SLEs and ML induced SLEs, no significant differences are observed using the cluster permutation technique. The results are consistent with the PSD shown in Figure 5, where ML TI and ML implantable induced equivalent beta/gamma oscillations and SLEs compared

to the baseline recorded. Additionally, control stimulations using only the envelope frequency applied to the cortex show no significant difference to baseline using t-score (not shown).

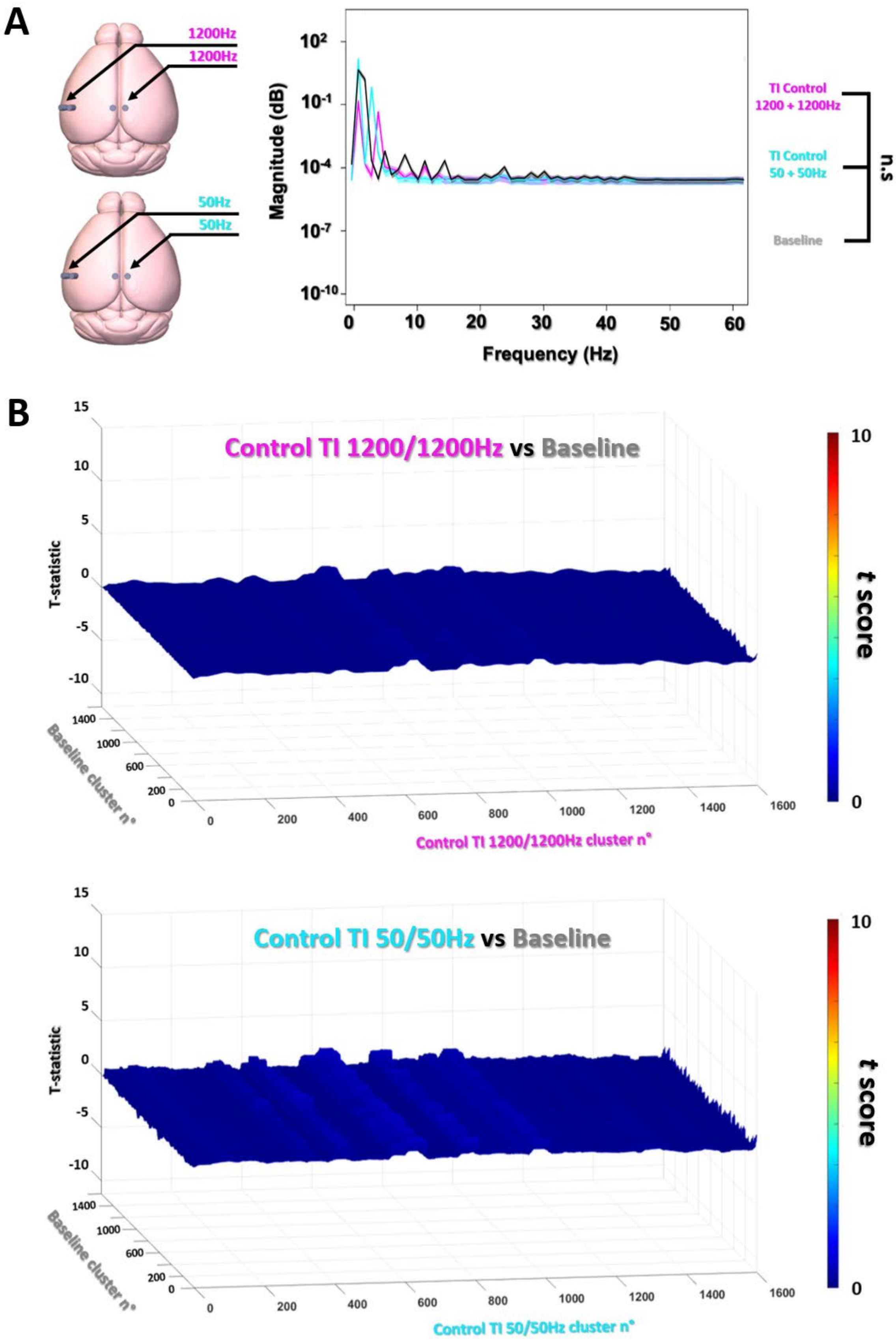
A
1200Hz
1200Hz
50Hz
50Hz
Magnitude (dB)
Frequency (Hz)
TI Control
1200 + 1200Hz
TI Control
50 + 50Hz
Baseline
n.s
B
Control TI 1200/1200Hz vs Baseline
T-statistic
Baseline cluster n°
Control TI 1200/1200Hz cluster n°
t score
Control TI 50/50Hz vs Baseline
T-statistic
Baseline cluster n°
Control TI 50/50Hz cluster n°
t score

**SUPPLEMENTARY FIGURE S9 | Control experiments for TI stimulation. (A)** There is no difference in electrophysiological activity recorded in the hippocampus between baseline data, cortical stimulation at 50 Hz, or cortical stimulation at 1200 Hz (no envelope). Each group is n = 4. **(B)** Similar cluster-based analysis as seen in Supplementary Figure 8 ((cluster size = 20ms). The t-score for recordings in the hippocampus during low-frequency stimulation provided to the cortex compared to baseline recordings in the hippocampus show no significance.
